# Genome-Wide Differential Expression Profiling of Pulmonary circRNAs Associated With Immune Reaction to *Pasteurella multocida* in Goats

**DOI:** 10.3389/fvets.2021.615405

**Published:** 2021-06-21

**Authors:** Qiaoling Chen, Zhenxing Zhang, Si Chen, Jie Chen, Yiwen Cheng, Ang Liu, Bin Li, Zhen Chen, Yiying Zheng, Manchuriga Ga, Li Du, Fengyang Wang

**Affiliations:** Hainan Key Lab of Tropical Animal Reproduction & Breeding and Epidemic Disease Research, College of Animal Science and Technology, Hainan University, Haikou, China

**Keywords:** *Pasteurella multocida*, lung, goat, CircRNA, ceRNA

## Abstract

*Pasteurella multocida* is a highly versatile pathogen that infects a wide range of animals, including goats, causing pneumonia and hemorrhagic septicemia. Circular RNA (circRNA) is a type of non-coding RNA that plays an important role in regulating cellular metabolism. However, whether and how circRNA is involved in regulating immune responses in the goat lung has not been reported. Thus, this study was designed to examine the function of circRNA in goats infected with *Pasteurella multocida*. Goats were assigned into one of two groups: an uninfected control group (CK) and an infected group challenged with *P. multocida*. Compared with the CK group, which remained healthy, the infected goats showed clinical signs of infection, including depression, cough, nasal discharge, and dyspnea, along with elevated body temperature and lesions in the lung. Whole-transcriptome sequencing and small RNA sequencing were then performed using lung samples from goats from each group. A total of 138 circRNA, 56 microRNAs (miRNA), and 2,673 messenger RNA (mRNA) molecules were significantly differentially expressed in the *P. multocida*-infected group compared with the CK group. Randomly selected differentially expressed circRNA, miRNA, and mRNA molecules (*n* = 5 per group) were then validated by quantitative reverse-transcriptase polymerase chain reaction analysis. Gene ontology (GO) analysis of the source genes indicated that six immune-related terms were enriched among the differentially expressed cirRNA molecules, including inflammatory response, immune effector process, cell activation involved in immune response, cytokine-mediated signaling pathway, response to endogenous stimulus, and immune response. The corresponding circRNA molecules were then selected for construction of a competitive endogenous RNA network to identify networks that may be involved in the immune response to *P. multocida* infection. The results indicated that *P. multocida* HN01 may cause pneumonia and stimulate an immune response in goats via regulation of circRNA expression. This study presents the first comprehensive circRNA profile in response to *P. multocida* infection in goats, thus, providing a basis for understanding the function of circRNA in the host immune response to *P. multocida* infection.

## Introduction

*Pasteurella multocida* is a Gram-negative bacterium that infects a wide range of domestic and wild animals. In addition, *P. multocida* causes zoonotic infection in humans, generally as a result of a bite from a cat or dog (1). As an opportunistic pathogen, *P. multocida* is associated with both chronic and acute infections that result in significant morbidity, with typical clinical signs including pneumonia, atrophic rhinitis, dermonecrosis, cellulitis, abscesses, meningitis, hemorrhagic septicemia, and even death, particularly in animals (2). Pasteurellosis, the general term for *P. multocida* infection, is one of the most important respiratory diseases in animals worldwide, with the associated mortality, reduced weight gain, and increased treatment costs resulting in significant economic losses in animal husbandry industries worldwide. Globally, pasteurellosis is one of the most common diseases of goats, with outbreaks usually leading to high mortality and economic losses. However, the pathogenesis of *P. multocida* infection in goats is not clear. To address this knowledge gap, goats were used as a model in the current study to explore the host reaction during *P. multocida* infection.

*P. multocida* contains multiple virulence factors, including lipopolysaccharides (LPS), capsule, toxin, adhesins, outer membrane proteins, and porins, all of which contribute to the fitness and pathogenicity of *P. multocida* (3). These virulence factors are indispensable for the pathogenesis of *P. multocida* and appear to be involved in the process of host invasion as well as survival and replication within the host. Among the virulence factors, the capsule and LPS constitute the major components of the bacterial cell surface and play crucial roles in the pathogenesis of *P. multocida*. The virulence of capsular mutants of different serogroups of *P. multocida* was found to be strongly attenuated in mice (4, 5). LPS functions in the bacterial membrane barrier and strongly interacts with the host immunity, thereby, playing a crucial role in the interaction between the bacteria and the environment (3). LPS has also been demonstrated with a direct role in defending the organism against the avian innate immune system (6). Protein toxin is another virulence factor that can affect the immunological response of the host's immune system. A new study indicated that Pm0442, a lipoprotein of *P. multocida*, affects the synthesis of capsular protein and LPS, and mediates the pro-inflammatory cytokine production of host macrophages (7). In spite of this finding in support of the virulence factor of *P. multocida* interaction with the host immune system, the exact molecule mechanisms taking place in the immune system of the host are still mostly unknown for these virulence factors of *P. multocida*. Therefore, the transcripts of the goat host were explored in this study for the basis study of pathogenicity of *P. multocida*.

Circular RNA (circRNA) is a novel type of non-coding endogenous RNA that is abundantly expressed in eukaryotes and was first described in bacterial pathogens in 1976 (8). CircRNAs are closed continuous loop structures with joined 3′ and 5′ ends by backsplicing of a single pre-mRNA molecule and more stable than other species of linear RNAs *in vivo* (5). However, an in-depth study of the function of circRNA only began in about 2010 with the advancement of RNA-seq technologies and the development of bioinformatic methods and programs (9). The resulting studies have reported that circRNA functions as a microRNA sponge, RNA-binding protein sequestering agent, and nuclear transcriptional regulator, suggesting that circRNA may be a component of the regulatory networks governing gene expression in transcriptional, posttranscriptional, and translational regulation. Thus, circRNAs participate in the regulation of physiological and pathological processes (10, 11). Moreover, disordered circRNA expression has been implicated in several diseases (12–14), making circRNA molecules effective biomarkers for the diagnosis and treatment of disease. However, circRNA participation in biological processes remains mostly undescribed. In a present study, we hypothesized that *P. multocida* infection could change the physiology and transcripts including circRNA expression in the lungs of goats. In order to validate this hypothesis, we performed and analyzed goat lung transcript level change during the *P. multocida* infection.

## Materials and Methods

### Bacterial Strain Preparation

*P. multocida* strain HN01 was previously isolated by our laboratory from lung samples collected from Hainan Black goats that died of pneumonia in Hainan Province, China (GenBank accession No. cp037861). Species designation was confirmed by 16S rRNA gene sequencing and comparison against the GenBank database. For the current study, *P. multocida* HN01 isolates were revived from storage at −80°C and cultured on blood agar at 37°C for 24 h. Uniform colonies were selected and further cultured in tryptic soy broth supplemented with 5% (v/v) bovine serum at 37°C overnight for 12 h with shaking prior to use in assays. For the challenge assays, *P. multocida* HN01 concentrations [colony-forming units (CFUs) ml^−1^] were first determined by plating serial dilutions on plate count agar. Goats were then intratracheally infected with 1 × 10^9^ CFUs of *P. multocida* HN01 in 2 ml of sterilized phosphate-buffered saline (PBS, pH 7.2).

### Animals

A total of eight clinically healthy 3 month-old male Hainan Black goats with 18 ± 2 kg weight were purchased from Huarun Goat Farm in Wanning City, Hainan Province, China. Nasal swabs and blood were collected every 3 days for at least 2 weeks prior to purchase to ensure that the goats were free of *P. multocida* and *Brucella* infection. The animals were then transported by road to the experimental station, where they were divided randomly into two groups (*n* = 4 per group): (1) an uninfected control group (CK) and (2) an infected group challenged with *P. multocida* (Pm). All goats were housed in separate sterile pens with age-appropriate temperature and humidity levels, and fed a typical standard control diet with access to both food and water *ad libitum*.

### Challenge Assay and Sample Collection

Goats in the challenge group were infected via intratrachea inoculation with 2 ml (1 × 10^9^ CFU) of *P. multocida* HN01 bacterial suspension, while goats in the CK group were inoculated with 2 ml of PBS 1 week after the animals' arrival. The inoculant concentration and assay methodology were as described previously (15). Goats were moved to fresh pens prior to challenge and were closely monitored throughout the experimental period for clinical signs of infection. Rectal temperatures and blood samples were collected daily from each goat during the study period to detect *P. multocida*. After the challenge assay, cumulative clinical scores (CCS) of all animals were obtained by using a score system introduced by Kacar et al., including respiratory rate, lung auscultation findings, laryngotracheal palpation results, and rectal temperatures (Supplementary Table 1) (16). One of the challenged goats died from acute disease within 8 h postinoculation, and other goats were euthanized 3 days post-*P. multocida* challenge. Lung tissue containing lesions was collected from each goat in the Pm group and from the corresponding regions of the lungs of goats from the CK group, and divided into two aliquots. One aliquot was stored in liquid nitrogen at −80°C until RNA extraction, while the other was fixed in 4% (v/v) paraformaldehyde for pathological examination following hematoxylin and eosin staining. In the meantime, one part of the liver and spleen were collected and fixed in 4% paraformaldehyde for histopathological analysis.

All experimental protocols were approved by the Academic Committee of the College of Animal Science and Technology of Hainan University based on the regulations on the use of the experimental animals and the institutional safety procedures.

### RNA Extraction, Library Preparation, and Sequencing

Total RNA was extracted from goat lung tissue using Trizol reagent (Takara Bio, Kusatsu, Japan) according to the manufacturer's protocol. RNA integrity and concentration were determined using an Agilent 2100 bioanalyzer (Agilent Technologies, Santa Clara, CA, USA) and detected by agarose gel electrophoresis. rRNA was removed from total RNA samples using a Ribo-Zero rRNA Removal Kit (Illumina, San Diego, CA, USA) and treated with RNase R (Illumina, San Diego, CA, USA) per the manufacturer's instructions. Three lung RNA samples from three animals per treatment were used for transcriptome sequencing. Ribosome-depleted RNA was fragmented and reverse-transcribed into first- and second-strand cDNA using an mRNA-Seq Sample Preparation Kit (Illumina) as per the manufacturer's instructions. The cDNA libraries were then evaluated by the Bioanalyzer 2000 and paired-end sequenced using a next-generation sequencing platform (Personalbio, Nanjing, China).

Small RNA libraries were constructed using a TruSeq Small RNA Preparation Kit (Illumina). Sizes were selected by polyacrylamide gel electrophoresis and processed for small RNA sequencing by Personalbio using the Illumina platform. All RNA and small RNA raw sequencing data generated in this work have been deposited in the NIH short-read archive under accession numbers PRJNA656854 and PRJNA656857, respectively.

### Identification of Differentially Expressed circRNA, miRNA, and mRNA Molecules

To identify differences in the expression of mRNA among samples, the raw sequencing data were filtered using Cutadapt (v2.7) (17) to retain high-quality reads for further analysis. The filtered reads were mapped to the reference genome (http://asia.ensembl.org/Capra_hircus/Info/Index) using HIsat2 (v2.1.0) (18) with the default mismatch of ≤2, and the alignment region distribution of the mapped reads was calculated. The HTSeq (v0.11.1) (19) statistic was used to compare read count values for each mRNA transcript to determine the original mRNA expression level, with fragments per kilobase of transcript per million (FPKM) mapped read values then used to standardize gene expression. After alignment with the reference genome, for the unmapped reads with the genome, 20 bp of each end was intercepted and used for anchor reads. These anchor reads were mapped to the reference genome by the Bowtie2 (20) software, and then the mapped data were used for identifying circRNA by find_circ with the suggested settings (21). The identification results of circRNA read count were used as the original expression level of circRNA and normalized to transcripts per million to measure the expression level.

Small RNA raw sequencing data were processed to filter out the low-quality data. Then the number of clean reads with a sequence length of 18–36 nt was counted. The exact same sequences in a single sample were de-reprocessed, and the sequence abundance was counted. These sequences obtained called “unique reads,” were used for subsequent analysis. The unique reads were then aligned to the goat genome using miRDeep2 (22) to obtain the distribution of small RNA on the genome. The obtained small RNA reads were compared with miRBase, piRBase, and Rfam to identify the conserved miRNAs, piRNAs, and other non-coding RNAs. The results of each conserved miRNA abundance count in each sample were normalized to FPKM.

Differentially expressed mRNA, circRNA, and miRNA molecules were defined as those with a *p* < 0.05 and |log_2_FC| > 1, as determined by the DESeq (23) analysis. The heatmap of differentially expressed mRNA, circRNA, and miRNA molecules was constructed using Pheatmap soft package in R language (distance measure using Euclidean and clustering algorithm using complete linkage). A heatmap was generated to show the differences in the expression of each gene among different samples The Fisher's exact test was used, and a *p* < 0.05 was used as the cut-off criterion for GO and Kyoto Encyclopedia of Genes and Genomes (KEGG) pathway analyses.

### Validation of circRNA, miRNA, and mRNA Molecules by Quantitative Reverse-Transcriptase Polymerase Chain Reaction (qPCR) Analysis

Total RNA was extracted from tissue samples using Trizol reagent (Invitrogen, Carlsbad, CA, USA) according to the manufacturer's instructions. RNA quantity and quality were measured using a NanoDrop 2000 spectrophotometer (Thermo Fisher Scientific, Waltham, MA, USA), while RNA integrity was assessed by standard denaturing agarose gel electrophoresis. RNA was then reverse-transcribed using a PrimeScript 1st Strand cDNA Synthesis Kit (Takara Bio, Kusatsu, Japan), and the resulting cDNA was used in qPCR assays to examine circRNA, mRNA, and miRNA expression. All qPCR assays were performed using a final reaction volume of 20 μl with SYBR Green PCR Master Mix (Takara Bio) according to the manufacturer's instructions in a Bio-Rad CFX-96 Touch instrument (Bio-Rad Laboratories, Hercules, CA, USA). Primers for mRNA and circRNA amplification were designed using Primer Premier 5.0 software (PRIMER Biosoft International, Palo Alto, CA, USA) and are shown in Supplementary Table 2. The thermal cycler parameters were as follows: 95°C for 30 s, followed by 40 cycles of 94°C for 10 s, primer-specific annealing temperature for 30 s, and 72°C for 10 s. MiRNA expression was detected using TaqMan probe-based qPCR, as described previously (24). The primers and TaqMan probes used for qPCR assays were designed using Beacon Designer 7 (PREMIER Biosoft International, Palo Alto, CA, USA). Detailed information on the primers and probes is provided in Supplementary Table 2. The reaction mixtures used for the qPCR assays contained 1 μl of cDNA, probe (200 nM), 0.4 μl of ROX, 10 μl of 2× AceQ U+ Universal Probe Master Mix (Vazyme, Hangzhou, China), forward and reverse primers (400 nM), and 8.6 μl of sterile water. All reactions were carried out at 95°C for 5 min, followed by 40 cycles at 95°C for 15 s and 60°C for 30 s. The relative expression of the target genes was calculated using the 2^−ΔΔCt^ method and normalized to the expression of the *GAPDH* (circRNA and mRNA molecules) and *5S rRNA* (miRNA molecules) genes. Results were expressed as fold-change relative to the average value of the control group. All experiments were conducted independently three times.

### Bioinformatics Analysis and Competing Endogenous RNA (ceRNA) Network Construction

To explore the function of circRNA, a ceRNA network was constructed using Cytoscape v3.6.0 (25). Potential circRNA–miRNA and miRNA–mRNA interactions were predicted using miRanda (http://miranda.org.uk/). miRanda max free energy values <10 and scores >140 were defined as the cutoff points for target prediction. Correlations between circRNA and miRNA expression levels were calculated using an SPSS Pearson correlation assay (Chicago, IL, USA).

### Statistical Analysis

All data were processed with GraphPad Prism 6.0 and presented as mean ± standard error of the mean (SEM). The cumulative clinical score of the goats was subjected to the non-parametric Mann–Whitney U-test by using R-language, and *p* < 0.05 was defined as statistically significant.

## Results

### Clinical Observations

One of the challenged goats died from acute disease within 8 h of inoculation and was visibly foaming at the mouth. All remaining challenged goats survived until the end of the 3 day experimental period but displayed clinical signs of infection including depression, loss of appetite, cough, nasal discharge, and dyspnea. In addition, a rectal temperature >40°C was observed among goats in the Pm group, while goats in the CK group had an average rectal temperature of 39°C. The CCS was increased significantly after the Pm HN01 challenge (*p* = 0.03191, *p* = 0.03191, and *p* = 0.03007 after 1, 2, and 3 days of inoculation with Pm HN01, respectively).

### PCR-Based Detection of *Pasteurella multocida* From Blood and Serum Swabs

To assess whether *P. multocida* persisted in the goats following inoculation, serum samples were collected from each goat for PCR-based detection of *P. multocida*. As shown in Supplementary Figure 1, *P. multocida* was detected in the serum of two challenged goats but was not detected in the serum samples from goats belonging to the CK group.

### Histopathological Analysis

**Figure 1:**
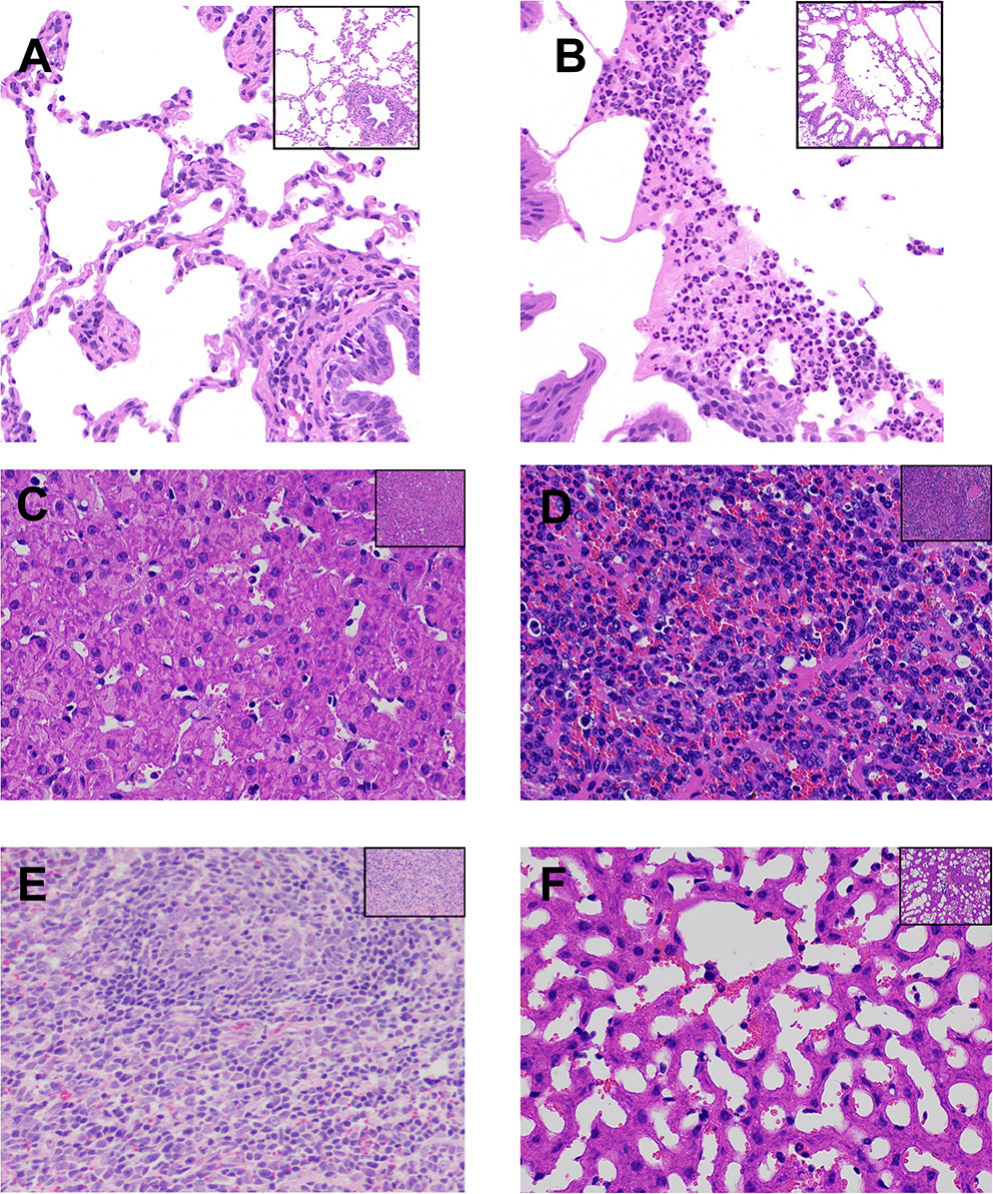
Histopathological analysis of lesions in the lungs, liver, and spleen of goats from the two experimental groups. **(A,C,E)** Lung, liver, and spleen tissues, respectively, from goats belonging to the uninfected control (CK) group. **(B D,F)** Lung, liver, and spleen tissues, respectively, from goats belonging to the *Pasteurella multocida*-challenged group. All tissues were strained with hematoxylin and eosin. Magnification, ×200 in the top right corner figure and ×400 in the larger-version figure. **(B)** Shows that some exfoliated alveolar epithelial cells and a large number of neutrophils gathered in the alveolar cavity, and some neutrophils degenerated and necrotized, forming abscess. **(D)** Shows that the normal structure of the liver tissue was lost, and the sinusoidal space was highly dilated and vacuolated, which compressed the hepatocyte cord and atrophied, and some hepatocytes were degenerated and necrotized. **(F)** Shows that lymphocyte proliferation and congestion could be seen in the spleen tissue, showing the characteristics of acute splenitis.

The morphology of the lungs and other organs was monitored to assess the pathological effects of *P. multocida* infection (Figure 1). The gross lesions in the challenged goats resulted in localized areas of lung edema, pulmonary hyperemia and hemorrhage, and degeneration of liver tissue. The boundary between the renal cortex and medulla was no longer defined in the *P. multocida*-challenged goats, and localized hemorrhaging was noted in some lymph nodes. Histological analysis of the lung, liver, and spleen tissues revealed varying degrees of damage as a result of *P. multocida* infection (Figure 1). Some exfoliated alveolar epithelial cells and a large number of neutrophils were observed in the alveolar cavity, and some neutrophils degenerated, necrotized, and formed abscesses (Figure 1B). The normal structure of the liver tissue was lost, and the sinusoidal space was highly dilated and vacuolated, squeezing the hepatocyte cord to atrophy. Some hepatocytes were degenerated and necrotized (Figure 1D). Lymphocyte proliferation and congestion were evident in the spleen tissue, showing the characteristics of acute splenitis, with heavy neutrophil infiltration in the spleen tissue (Figure 1F). In contrast, no pathological changes were observed in the lung, liver, and spleen tissues of goats belonging to the CK group.

### Differentially Expressed circRNA, miRNA, and mRNA Molecules

To determine the effects of *P. multocida* on RNA expression in the lungs of goats, the expression of circRNA, mRNA, and miRNA was examined in the CK and Pm groups. A total of 138 circRNA, 56 miRNA, and 2,673 mRNA transcripts were significantly differentially expressed in the Pm group compared with the CK group (*p* < 0.05). A heatmap was then generated to visualize the differentially expressed RNA molecules (Supplementary Figure 2 and Supplementary Table 3). In total, 89 and 49 circRNA molecules were up- and down-regulated, respectively, in the Pm group, while 42 and 14 miRNA molecules were up- and down-regulated, respectively (Supplementary Figures 2A–C). Overall, 1,305 mRNA molecules were upregulated and 1,368 were downregulated in the Pm group compared with the CK group (Supplementary Figures 2A,D).

### qPCR Validation

**Figure 2:**
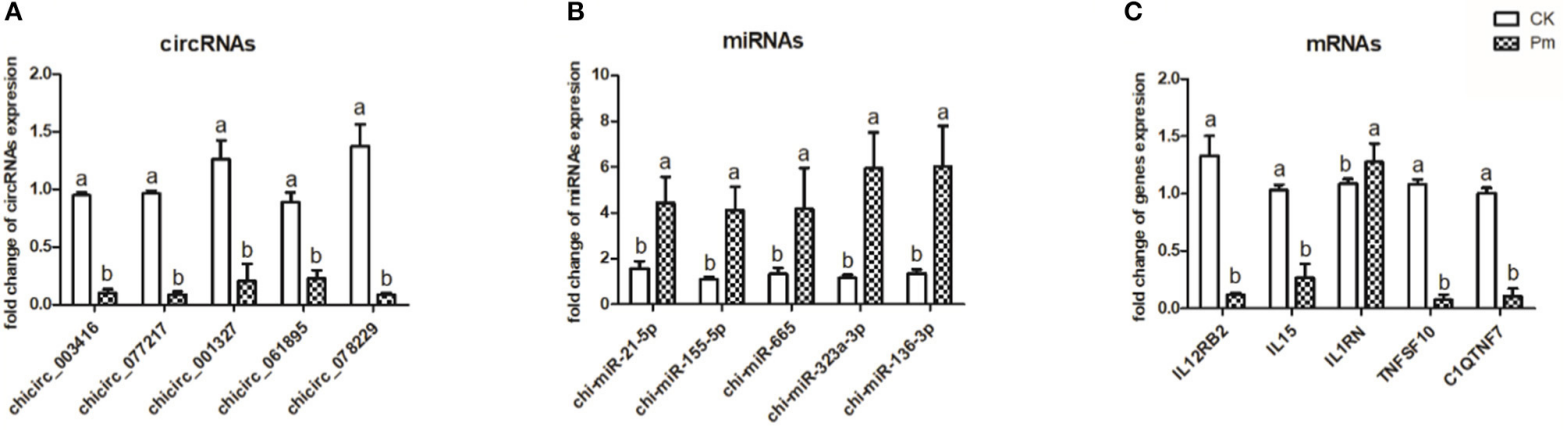
Expression of five randomly selected circRNA **(A)**, miRNA **(B)**, and mRNA **(C)** molecules in the goat lung samples as determined by qPCR analysis. *GAPDH* was used as a reference for normalization of mRNA and circRNA expression, while *5S rRNA* was used as a reference for normalization of miRNA expression. The different letters above the bars indicate significant differences among groups (*p* < 0.05). Samples were collected from three goats per group (CK, uninfected control group; Pm, *P. multocida*-challenged group).

To confirm and quantify the differentially expressed circRNA, miRNA, and mRNA molecules in the CK and *P. multocida* samples, five of each type were randomly selected and validated by qPCR using the lung samples from each group (Figure 2). The results confirmed that the RNA expression profiles were consistent with the RNA-Seq results.

### Gene Ontology and Kyoto Encyclopedia of Genes and Genomes Analysis of circRNA and mRNA Molecules

**Figure 3:**
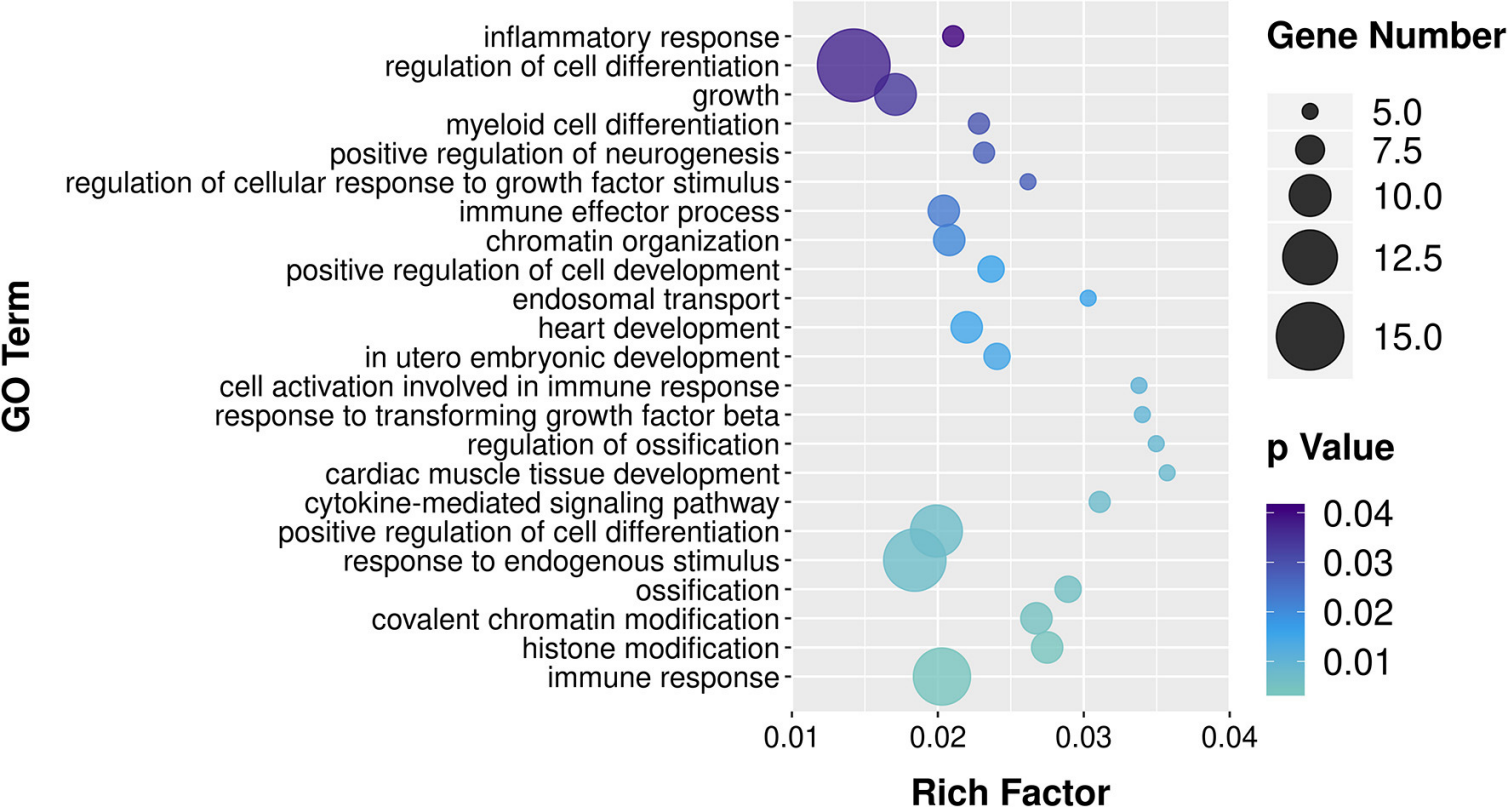
Enriched biological process Gene Ontology (GO) terms among the source genes of differentially expressed circRNA molecules. The x-axis indicates the enrichment factor of the GO terms, while the names of the GO terms are shown on the y-axis. The size and color of the dot indicate the number of source genes and the *p*-value (Fisher's exact test), respectively. The enrichment factor indicates the number of differentially expressed genes/total genes for each GO term.

**Figure 4:**
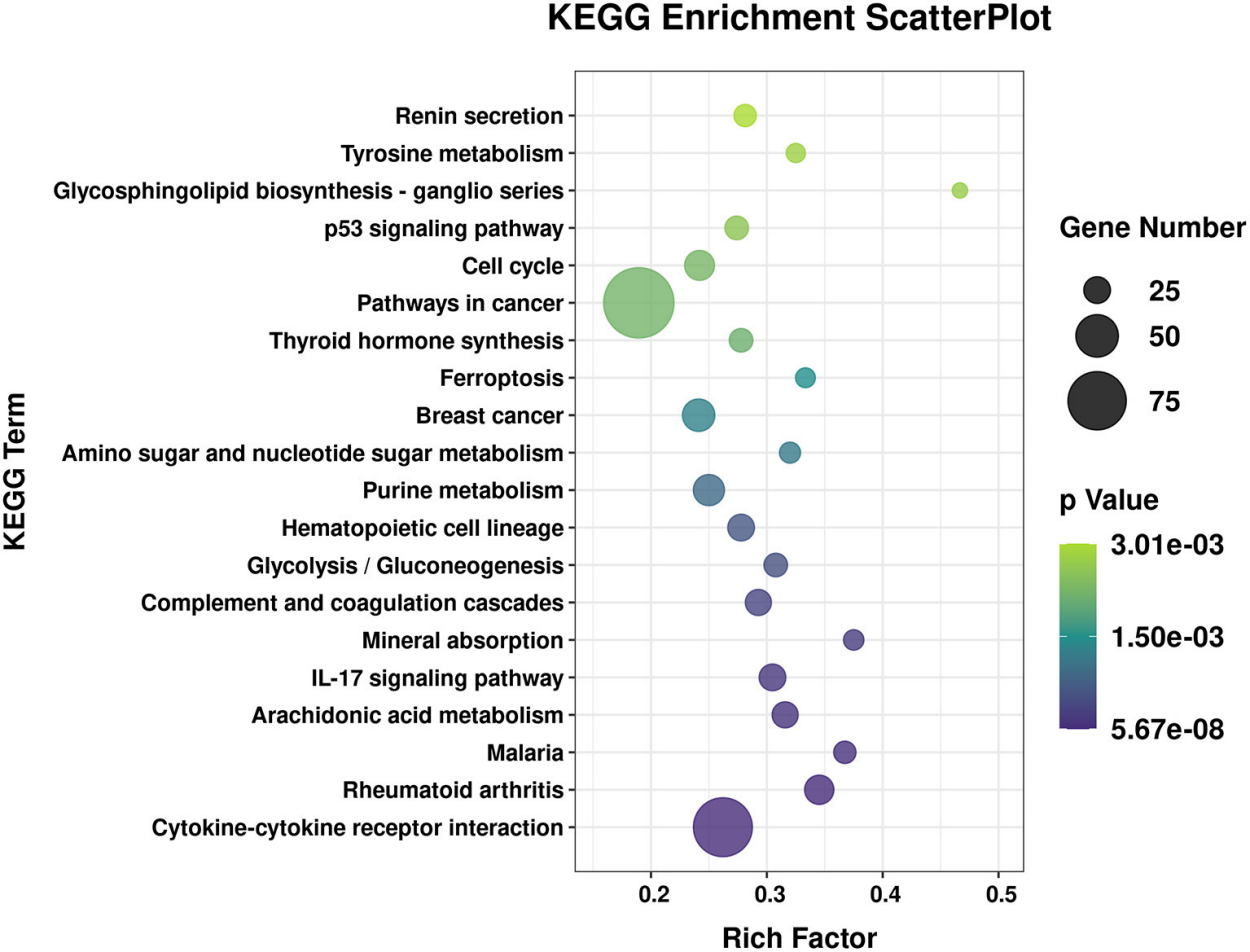
Top 20 of the enriched Kyoto Encyclopedia of Genes and Genomes (KEGG) pathways of the differentially expressed RNA molecules. The x-axis indicates the enrichment factor, and the y-axis indicates the name of the KEGG pathway. The size and color of the dot indicate the number of target genes and the *p*-value (Fisher's exact test), respectively. The enrichment factor indicates the number of differentially expressed genes/total genes for each KEGG pathway.

To explore the effects of *P. multocida* on lung function in goats, the source genes of the differentially expressed circRNA molecules and mRNA were subjected to GO and KEGG analyses. The enriched KEGG pathway circRNA molecules are listed in Supplementary Table 4. Some metabolism in the KEGG pathway were enriched, such as amino sugar and nucleotide sugar metabolism, fructose and mannose metabolism, glycerophospholipid metabolism, etc. The GO term with both more than four circRNA molecule source gene list and a *p* < 0.05 is shown in Supplementary Table 5 and the biological process GO terms are displayed in Figure 3. A large number of the enriched GO terms were associated with immune response-related biological processes, including inflammatory response, immune effector process, cell activation involved in immune response, cytokine-mediated signaling pathway, response to endogenous stimulus, and immune response. The enriched GO and KEGG of the differentially expressed RNA molecules were also analyzed and listed in Supplementary Tables 6, 7 respectively. The top 20 of the KEGG pathways are presented in Figure 4. Among the enriched pathways, the p53 signaling pathway, IL-17 signaling pathway, and cytokine–cytokine receptor interaction immune pathways are listed in the top 20 of the KEGG pathways of DGEs.

### ceRNA Network Construction

**Figure 5:**
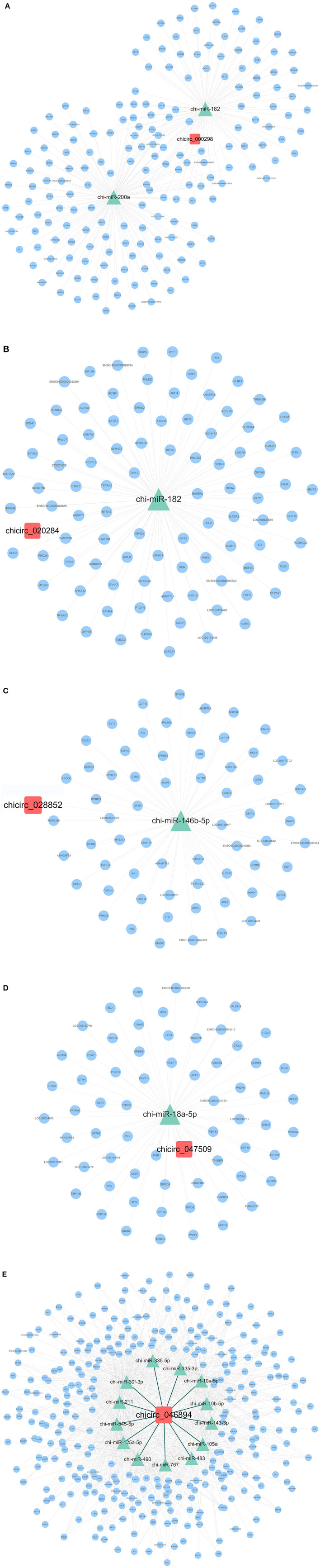
The ceRNA network of the four downregulated **(A–D)** and one upregulated (chicirc_04689) **(E)** circRNA molecules. Red squares, green triangles, and blue circles indicate the differentially expressed circRNA, miRNA, and mRNA molecules, respectively.

**Figure 6:**
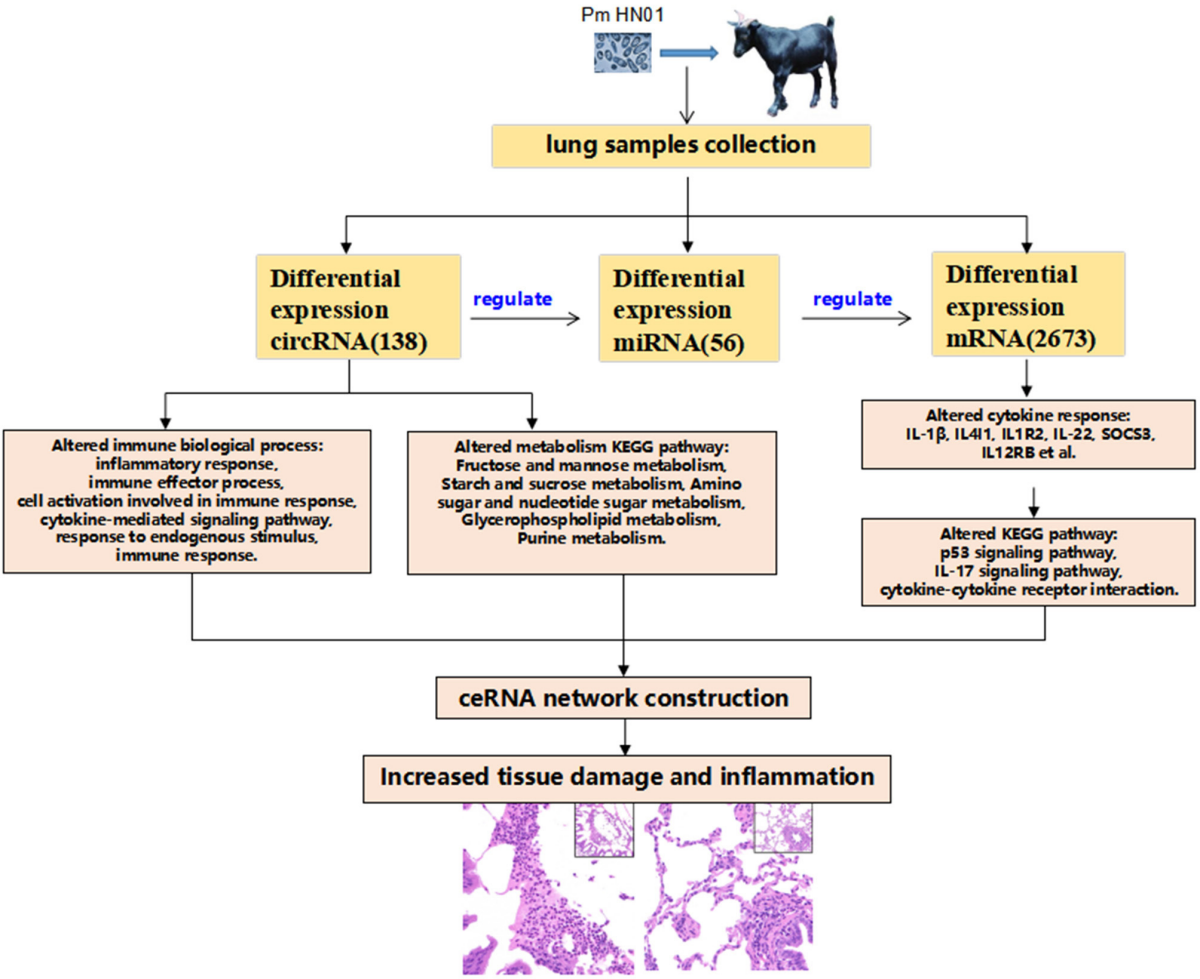
Schematic overview of the study results. *P. multocida* was used to challenge goats, which caused pathological changes in tissues. After transcriptome sequencing with the lungs, the transcriptome expression level of lung tissues were changed significantly. Differentially expressed circRNAs were involved in host immunology biological processes and metabolic pathways. circRNAs function as a miRNA sponge, regulated the expression of miRNAs, and miRNAs regulated a wide range of target genes forming the circRNA–miRNA–mRNA regulating network. A large number of differentially expressed cytokines [interleukin-1beta (IL-1β), interleukin-1beta (IL4I1), interleukin 1 receptor type 2 (IL1R2), IL-22, suppressor of cytokine signaling 3 (SOCS3), and (IL12RB)] participate in the host's response to the invasion of external pathogens (Pm HN01), causing inflammatory response and pathological changes in the lung tissues of goats.

A total of 26 circRNA source genes were enriched in six immune-related biological processes. Compared with the CK group, 7 and 19 circRNA molecules were down- and upregulated, respectively, in the Pm treatment group. The target miRNA transcripts of the 26 circRNA molecules were then predicted, along with the miRNA target genes. ceRNA networks were then constructed for the 26 circRNA molecules based on upregulated circRNA molecules, downregulated miRNA molecules and upregulated genes or downregulated circRNA molecules, and up-regulated miRNA molecules and downregulated genes, respectively, to further explore the biological functions of the 26 differentially expressed circRNA molecules during *P. multocida* infection. Overall, 4 downregulated circRNA and 11 upregulated circRNA molecules were placed into ceRNA networks (Supplementary Table 8). Based on the criteria used in this study, the corresponding regulatory miRNA was not determined for the remaining circRNA molecules. The ceRNA network for the four downregulated circRNA molecules (chicirc_000298, chicirc_020284, chicirc_028852, and chicirc_047509) is shown in Figures 5A–D. Among the 11 upregulated circRNA molecules, chicirc_04689 had the largest number of target miRNA transcripts, with 13 miRNA molecules were identified as being downregulated by chicirc_04689. The target genes for these 13 downregulated miRNA molecules were then determined, and differences in their expression between the two treatment groups were assessed. In total, 939 genes were identified as being significantly upregulated in the Pm group compared with the CK group (Figure 5E). Finally, the summary and profile of the results were merged and are shown in Figure 6.

## Discussion

The *P. multocida* strain used in this study was originally isolated from goats. Peng et al. (26) reported that there are few available *P. multocida* isolates from goats and that, in general, *P. multocida* strains demonstrate greater pathogenicity in their original host species (27). In another study, different *P. multocida* strains from various hosts were used to infect goats. Goats challenged with 1.6 × 10^8^ CFU of a caprine *P. multocida* type D strain all died within 8 days of infection, while at least some of the goats challenged with *P. multocida* strains originally isolated from other species survived until the end of the 14 day study period (15). Thus, *P. multocida* HN01 was used in the current study to explore the mechanism of *P. multocida* pathogenicity in goats. Following HN01 infection, one of the challenged goats died within the first 8 h as a result of an acute reaction, while the three remaining challenged goats showed increased body temperature and clinical signs of respiratory tract infection compared with the control animals. Zamri-Saad et al. (15) established a pneumonic pasteurellosis model in goats, with the infected animals displaying similar clinical symptoms to those in the current study. In addition, *P. multocida*-challenged pigs and chickens also showed elevated body temperature and labored breathing (27, 28). In all studies, control animals showed no clinical signs of disease. In the current study, *P. multocida* was detected in the serum of two goats from the experimental group, indicating that bacteria had entered the bloodstream by 3 days postinoculation.

*Pasteurella* inoculation caused pneumonia in the infected goats in the current study (Figure 1) as a result of pneumonic lesions and microscopic tissue damage. *P. multocida* predominantly causes hemorrhagic septicemia and respiratory disease in ruminant and hoofed animals (28). The symptoms of pneumonic pasteurellosis described in the literature (15, 29) are in line with the lung changes and clinical symptoms observed in goats in the current study. In addition, other organs such as the liver and spleen also showed an immune response reaction to *P. multocida* infection, with symptoms, and some hepatocytes were degenerated and necrotized in the liver tissue and the spleen tissue showing characteristics of acute splenitis (Figures 1D,F). In cases of pasteurellosis, the host immune response is caused by a number of *P. multocida* virulence factors that interact with the host's immune system. However, the exact molecular mechanisms taking place inside innate and adaptive immune cells in response to *P. multocida* virulence factors are largely unknown (2). The immune system is a highly regulated network of cellular interactions that protects an organism from the potential dangers of invading bacteria (30). However, our understanding of how the multiple immune organs and cells work together in goats to inhibit *P. multocida* infection is limited. Therefore, we used next-generation sequencing to study the molecular mechanisms of the goat lung tissue response to *P. multocida* invasion in an attempt to better understand transcript-level changes in the host.

Although the importance of RNA transcripts in host lung inflammation as a result of *P. multocida* infection in mice and chickens is well-known (31, 32), the molecular mechanisms underlying RNA-based regulation of the immune response in goats remain unclear. This study is the first to examine changes in RNA transcript expression in response to *P. multocida* infection in the goat lung. We identified a total of 138 circRNA, 56 miRNA, and 2,673 mRNA molecules that were significantly differentially expressed in *P. multocida*-challenged animals compared with the CK group and validated the results via qPCR analysis. Previously, Li et al. analyzed the transcriptome of chicken lungs infected with two isolates of *P. multocida* serotype A (PmCQ2 and PmQ-P) and identified differentially expressed genes (DEGs). In our study, we found 232 of the same DEGs as in the PmCQ2-infected lungs and 284 of the same DEGs as in the PmQ-P-infected chicken lungs. Interestingly, several of these DEGs [e.g., interleukin-1beta (IL-1β), interleukin 4-induced 1 (IL4I1), interleukin 1 receptor type 2 (IL1R2), IL-22, and suppressor of cytokine signaling 3 (SOCS3) cytokines] showed the same expression trend and were upregulated in *P. multocida*-infected lungs of goats and those of chicken. Through an analysis of the enriched KEGG pathways, we found that many of these same DEGs are involved in the p53 signaling pathway and cytokine–cytokine receptor interactions (33). The most enriched KEGG pathway cytokine–cytokine receptor interaction was also enriched by the mouse lung DGEs that infected with *P. multocida* (31). Together, these results indicate that *P. multocida* infection can induce the extensive cytokine changes that participated in the general inflammatory reaction of lungs of animals, including the mouse, chicken, and goat.

Among the differentially expressed mRNA-enriched pathways, there are 25 DEGs implicated in the IL-17 signaling pathway, of which only 2 were downregulated genes and 23 were upregulated. IL-17A has a similar biological function and 50% homology with IL-17F. Both are involved in the IL-17 signaling pathway and induce common transducing pathways through a common receptor with two chains: IL17RA and IL17RC (34, 35). In the current study, the differential expression of IL-17A (7.78log2FoldChange) was upregulated more than IL-17F (4.09log2FoldChange), which may be the reason why IL-17A has a stronger effect than IL-17F (35). IL-17A and IL-17F are key cytokines involved in the innate and adaptive immune response, and IL-17A induces the production of pro-inflammatory mediators of IL-6 in many types of cells (34). Accordingly, we also found that IL-6 was upregulated in *P. multocida*-infected lung tissue. IL-6 is a mediator of inflammation that plays critical roles in a broad range of immune responses, including pro-inflammatory responses and anti-inflammatory responses (36). In another study, IL-1β alone significantly increased IL-6 productions in different cells, with an additive effect for synoviocytes and a synergistic effect for hepatocytes with IL-17A and IL-17F (34). IL-1β was also upregulated in this study, which, therefore, suggests that IL-17A and IL-17F synergy with IL1β induces IL-6 production. These actions in the IL-17 signaling pathway may all contribute to the goat lung inflammation reaction and the overall host defense reaction to Pm HN01. Because dysregulation of the IL-17 pathway contributes to the immune pathology of the goat lungs, it will be important to further elucidate the molecular mechanisms governing the IL-17 signaling pathway in future studies.

GO analysis of the differentially expressed circRNA source genes revealed that several GO terms associated with immune-related biological processes were enriched, including inflammatory response, immune effector process, cell activation involved in immune response, cytokine-mediated signaling pathway, response to endogenous stimulus, and immune response (Figure 3). The results indicated an essential role for circRNA in supporting the fight against *P. multocida* infection and in triggering the host immune response in the goat lung.

We also constructed and analyzed 15 ceRNA networks generated from the differentially expressed transcripts (Supplementary Table 8), with chicirc_04689 showing the greatest number of target miRNA molecules. In total, 13 downregulated miRNA transcripts (chi-miR-335-3p, chi-miR-335-5p, chi-miR-143-3p, chi-miR-125a-5p, chi-miR-10a-5p, chi-miR-211, chi-miR-30f-3p, chi-miR-10b-5p, chi-miR-105a, chi-miR-483, chi-miR-545-5p, chi-miR-490, and chi-miR-767) were targeted by chicirc_04689. Studies on the function of these 13 miRNA molecules have mainly focused on various cancers, some of which have been researched extensively. Many studies have investigated the function of miR-221. In one review, the authors conducted a meta-analysis to investigate the significance of increased miR-221 expression; however, the results were inconclusive, which was attributed to the significant heterogeneity among systems and the limited number of studies for some cancers (37). Overexpression of miR-767-5p promoted cell progression by directly targeting and regulating MAPK4 (38), while miR-767-3p inhibited the growth and migration of lung adenocarcinoma cells by regulating CLDN18 (39). According to these studies, several miRNA transcripts targeted by chicirc_04689 prevent cancer progression by inhibiting cell migration, invasion, and proliferation. However, the expression of the 13 miRNA targets of chicirc_04689 was downregulated in the Pm infection group in the current study, which may be related to the *P. multocida* infection-induced host inflammation and immune response. The miRNA transcripts identified in this study also showed decreased expression in inflammatory lung tissues, which appears to inhibit the development of cellular inflammatory responses and is consistent with the literature. Of course, how these miRNA molecules are involved in regulating the host inflammatory response needs further study to confirm our current findings. In particular, how chicirc_04689 regulates the immune response by targeting these miRNA molecules deserves further attention.

*P. multocida* was used to challenge goats, which caused pathological changes in tissues. After transcriptome sequencing with lung, the transcriptome expression level of lung tissues was changed significantly. Differentially expressed circRNAs were involved in host immunology biological processes and metabolic pathways. circRNAS functions as a microRNA sponge, regulated the expression of miRNAs, and miRNAs regulated a wide range of target genes forming a circRNA–miRNA–mRNA regulating network. A large number of differentially expressed cytokines (IL-1β, IL4I1, IL1R2, IL-22, SOCS3, and IL12RB) participate in the host's response to the invasion of external pathogens (Pm HN01), causing inflammatory response and pathological changes in the lung tissue of the goat (Figure 6).

To our knowledge, the present study is the first comprehensive analysis of RNA transcript expression patterns in the lungs of goats infected with *P. multocida*. A total of 138 circRNA (89 upregulated and 49 downregulated), 56 miRNA, and 2,673 mRNA molecules were significantly differentially expressed in *P. multocida*-infected goats compared with the CK group. In addition, the infected animals showed typical clinical signs of pneumonia. GO analysis of the source genes of the differentially expressed circRNA molecules and KEGG pathway analysis of mRNA molecules showed that multiple biological processes and pathways associated with immune-related biological processes were significantly enriched, thus, allowing the potential roles of circRNA in regulating the host immune reaction in response to bacterial infection to be explored. However, the KEGG pathway analysis of differentially expressed circRNAs showed that they were enriched in some metabolism pathway of the host. The construction of ceRNA networks may co-participate in the inflammation and damage of lung tissue resulting from Pm HN01 infection and also provided new insights into the transcriptional landscape in the goat lung during infection by foreign bacteria.

## Data Availability Statement

All RNA and small RNA raw sequencing data generated in this work have been deposited in the NIH short read archive under accession numbers PRJNA656854 and PRJNA656857, respectively.

## Ethics Statement

The animal study was reviewed and approved by Academic Committee of College of Animal Science and Technology of Hainan University based on the regulations on the use of the experimental animals and the institutional safety procedures.

## Author Contributions

QC and FW conceived, designed, performed the experiments, analyzed the data, and prepared the manuscript. ZZ, SC, JC, YC, BL, and AL performed the animal experiments. ZC, YZ, AL, MG, and LD contributed reagents and prepared the material and figures and/or tables. All authors contributed to the article and approved the submitted version.

## Conflict of Interest

The authors declare that the research was conducted in the absence of any commercial or financial relationships that could be construed as a potential conflict of interest.

## Supplementary Material

Supplementary Figure 1 PCR-based validation of *P. multocida* in serum samples. Lanes 1–4, serum samples from the CK-group goats; lanes 5–7, serum samples from the Pm-group goats.

Supplementary Figure 2 Differentially-expressed circRNA, miRNA, and mRNA in the lung tissues of *P. multocida*-challenged goats compared with the CK group. **(A)** Numbers of differentially-expressed circRNA, miRNA, and mRNA molecules. **(B–D)** Hierarchical clustering analysis of differentially-expressed circRNA, miRNA, and mRNA molecules, respectively, identified at *p* < 0.05 in the goat lung.

Supplementary Table 1 Cumulative clinical scoring system.

Supplementary Table 2 Primers used for qPCR analyses.

Supplementary Table 3 List of differentially-expressed RNA molecules.

Supplementary Table 4 KEGG pathways enriched among source genes of differentially-expressed circRNA transcripts in goat lung tissue samples.

Supplementary Table 5 Gene ontology terms enriched among source genes of differentially-expressed circRNA transcripts in goat lung tissue samples.

Supplementary Table 6 Gene ontology terms enriched of differentially-expressed mRNA transcripts in goat lung tissue samples.

Supplementary Table 7 KEGG pathways enriched by differentially expressed mRNAs in goat lung tissue samples.

Supplementary Table 8 ceRNA network of differentially-expressed cirRNA transcripts.

## References

[1] Mogilner L Katz C. Pasteurella multocida. Pediatr Rev. (2019) 40:90–2. 10.1542/pir.2017-0178 30709978

[2] Wilson BA Ho M. *Pasteurella multocida*: from zoonosis to cellular microbiology. Clin Microbiol Rev. (2013) 26:631–55. 10.1128/CMR.00024-13 23824375 PMC3719492

[3] Harper M Boyce JD. The Myriad properties of *Pasteurella multocida* lipopolysaccharide. Toxins. (2017) 9:254. 10.3390/toxins9080254 28825691 PMC5577588

[4] Boyce JD Adler B. The capsule is a virulence determinant in the pathogenesis of *Pasteurella multocida* M1404 (B:2). Infect Immunity. (2000) 68:3463–8. 10.1128/IAI.68.6.3463-3468.2000 10816499 PMC97626

[5] Chung JY Wilkie I Boyce JD Townsend KM Frost AJ Ghoddusi M. Role of capsule in the pathogenesis of fowl cholera caused by *Pasteurella multocida* serogroup A. Infect Immunity. (2001) 69:2487–92. 10.1128/IAI.69.4.2487-2492.2001 11254611 PMC98183

[6] Harper M Boyce JD Adler B. The key surface components of *Pasteurella multocida*: capsule and lipopolysaccharide. Curr Topics Microbiol Immunol. (2012) 361:39–51. 10.1007/82_2012_202 22373812

[7] He F Qin X Xu N Li P Wu X Duan L. *Pasteurella multocida* Pm0442 affects virulence gene expression and targets TLR2 to induce inflammatory responses. Front Microbiol. (2020) 11:1972. 10.3389/fmicb.2020.01972 32922380 PMC7456837

[8] Sanger HL Klotz G Riesner D Gross HJ Kleinschmidt AK. Viroids are single-stranded covalently closed circular RNA molecules existing as highly base-paired rod-like structures. Proc Natl Acad Sci USA. (1976) 73:3852–6. 10.1073/pnas.73.11.3852 1069269 PMC431239

[9] Patop IL Wust S Kadener S. Past, present, and future of circRNAs. EMBO J. (2019) 38:e100836. 10.15252/embj.2018100836 31343080 PMC6694216

[10] Ebbesen KK Kjems J Hansen TB. Circular RNAs: identification, biogenesis and function. Biochim Biophys Acta. (2016) 1859:163–8. 10.1016/j.bbagrm.2015.07.007 26171810

[11] Guerra BS Lima J Araujo BHS Torres LB Santos JCC Machado DJS. Biogenesis of circular RNAs and their role in cellular and molecular phenotypes of neurological disorders. Semin Cell Dev Biol. (2020) 114:1–10. 10.1016/j.semcdb.2020.08.003 32893132

[12] Feng J Chen K Dong X Xu X Jin Y Zhang X. Genome-wide identification of cancer-specific alternative splicing in circRNA. Mol Cancer. (2019) 18:35. 10.1186/s12943-019-0996-0 30849979 PMC6408762

[13] Werfel S Nothjunge S Schwarzmayr T Strom TM Meitinger T Engelhardt S. Characterization of circular RNAs in human, mouse and rat hearts. J Mol Cell Cardiol. (2016) 98:103–7. 10.1016/j.yjmcc.2016.07.007 27476877

[14] Lukiw WJ. Circular RNA (circRNA) in Alzheimer's disease (AD). Front Genet. (2013) 4:307. 10.3389/fgene.2013.00307 24427167 PMC3875874

[15] Zamri-Saad M Effendy WM Maswati MA Salim N Sheikh-Omar AR. The goat as a model for studies of pneumonic pasteurellosis caused by *Pasteurella multocida*. Br Vet J. (1996) 152:453–8. 10.1016/S0007-1935(96)80039-X 8791853

[16] Kacar Y Batmaz H Yilmaz OE Mecitoglu Z. Comparing clinical effects of marbofloxacin and gamithromycin in goat kids with pneumonia. J South Afr Vet Assoc. (2018) 89:e1–5. 10.4102/jsava.v89i0.1558 29943581 PMC6138188

[17] Kechin A Boyarskikh U Kel A Filipenko M. cutPrimers: a new tool for accurate cutting of primers from reads of targeted next generation sequencing. J Comput Biol. (2017) 24:1138–43. 10.1089/cmb.2017.0096 28715235

[18] Kim D Langmead B Salzberg SL. HISAT: a fast spliced aligner with low memory requirements. Nat Methods. (2015) 12:357–60. 10.1038/nmeth.3317 25751142 PMC4655817

[19] Anders S Pyl PT Huber W. HTSeq–a Python framework to work with high-throughput sequencing data. Bioinformatics. (2015) 31:166–9. 10.1093/bioinformatics/btu638 25260700 PMC4287950

[20] Langmead B Salzberg SL. Fast gapped-read alignment with Bowtie 2. Nature Methods. (2012) 9:357–9. 10.1038/nmeth.1923 22388286 PMC3322381

[21] Memczak S Jens M Elefsinioti A Torti F Krueger J Rybak A. Circular RNAs are a large class of animal RNAs with regulatory potency. Nature. (2013) 495:333–8. 10.1038/nature11928 23446348

[22] Friedländer MR Mackowiak SD Li N Chen W Rajewsky N. miRDeep2 accurately identifies known and hundreds of novel microRNA genes in seven animal clades. Nucleic Acids Res. (2012) 40:37–52. 10.1093/nar/gkr688 21911355 PMC3245920

[23] Anders S Huber W. Differential expression analysis for sequence count data. Genome Biol. (2010) 11:R106. 10.1186/gb-2010-11-10-r106 20979621 PMC3218662

[24] Androvic P Valihrach L Elling J Sjoback R Kubista M. Two-tailed RT-qPCR: a novel method for highly accurate miRNA quantification. Nucleic Acids Res. (2017) 45:e144. 10.1093/nar/gkx588 28911110 PMC5587787

[25] Shannon P Markiel A Ozier O Baliga NS Wang JT Ramage D. Cytoscape: a software environment for integrated models of biomolecular interaction networks. Genome Res. (2003) 13:2498–504. 10.1101/gr.1239303 14597658 PMC403769

[26] Peng Z Liang W Wang F Xu Z Xie Z Lian Z. Genetic and phylogenetic characteristics of *Pasteurella multocida* isolates from different host species. Front Microbiol. (2018) 9:1408. 10.3389/fmicb.2018.01408 29997608 PMC6029419

[27] Peng Z Liang W Wang Y Liu W Zhang H Yu T. Experimental pathogenicity and complete genome characterization of a pig origin *Pasteurella multocida* serogroup F isolate HN07. Vet Microbiol. (2017) 198:23–33. 10.1016/j.vetmic.2016.11.028 28062004

[28] Zeng QL Mei X Su J Li XH Xiong WG Lu Y. Integrated pharmacokinetic-pharmacodynamic (PK/PD) model to evaluate the *in vivo* antimicrobial activity of Marbofloxacin against *Pasteurella multocida* in piglets. BMC Vet Res. (2017) 13:178. 10.1186/s12917-017-1099-z 28619095 PMC5471993

[29] Rawat N Gilhare VR Kushwaha KK Hattimare DD Khan FF Shende RK. Isolation and molecular characterization of *Mannheimia haemolytica* and *Pasteurella multocida* associated with pneumonia of goats in Chhattisgarh. Vet World. (2019) 12:331–6. 10.14202/vetworld.2019.331-336 31040578 PMC6460873

[30] Kubatzky KF. *Pasteurella multocida* and immune cells. Curr Topics Microbiol Immunol. (2012) 361:53–72. 10.1007/82_2012_204 22427181

[31] Wu C Qin X Li P Pan T Ren W Li N. Transcriptomic analysis on responses of murine lungs to *Pasteurella multocida* infection. Front Cell Infect Microbiol. (2017) 7:251. 10.3389/fcimb.2017.00251 28676843 PMC5476747

[32] Guterres A de Azeredo Lima CH Miranda RL Gadelha MR. What is the potential function of microRNAs as biomarkers and therapeutic targets in COVID-19? Infect Genet Evol. (2020) 85:104417. 10.1016/j.meegid.2020.104417 32526370 PMC7833518

[33] Li P He F Wu C Zhao G Hardwidge PR Li N. transcriptomic analysis of chicken lungs infected with avian and bovine *Pasteurella multocida* serotype A. Front Vet Sci. (2020) 7:452. 10.3389/fvets.2020.00452 32851030 PMC7433353

[34] Noack M Beringer A Miossec P. Additive or synergistic interactions between IL-17A or IL-17F and TNF or IL-1β depend on the cell type. Front Immunol. (2019) 10:1726. 10.3389/fimmu.2019.01726 31396230 PMC6664074

[35] Beringer A Noack M Miossec P. IL-17 in chronic inflammation: from discovery to targeting. Trends Mol Med. (2016) 22:230–41. 10.1016/j.molmed.2016.01.001 26837266

[36] Hasegawa H Mizoguchi I Chiba Y Ohashi M Xu M Yoshimoto T. Expanding diversity in molecular structures and functions of the IL-6/IL-12 heterodimeric cytokine family. Front Immunol. (2016) 7:479. 10.3389/fimmu.2016.00479 27867385 PMC5095122

[37] Ravegnini G Cargnin S Sammarini G Zanotti F Bermejo JL Hrelia P. Prognostic role of miR-221 and miR-222 expression in cancer patients: a systematic review and meta-analysis. Cancers. (2019) 11:970. 10.3390/cancers11070970 31336701 PMC6678869

[38] Feng Y Zhang L Wu J Khadka B Fang Z Gu J. CircRNA circ_0000190 inhibits the progression of multiple myeloma through modulating miR-767-5p/MAPK4 pathway. J Exp Clin Cancer Res. (2019) 38:54. 10.1186/s13046-019-1071-9 30728056 PMC6364482

[39] Wan YL Dai HJ Liu W Ma HT. miR-767-3p inhibits growth and migration of lung adenocarcinoma cells by regulating CLDN18. Oncol Res. (2018) 26:637–44. 10.3727/096504017X15112639918174 29169410 PMC7844711

