# Supplementary figures and images for: Genome-Wide Differential Expression Profiling of Pulmonary circRNAs Associated With Immune Reaction to Pasteurella multocida in Goats
Source: Front Vet Sci. 2021 Jun 21;8:615405. doi: 10.3389/fvets.2021.615405 (PMC8256745; doi:10.3389/fvets.2021.615405)

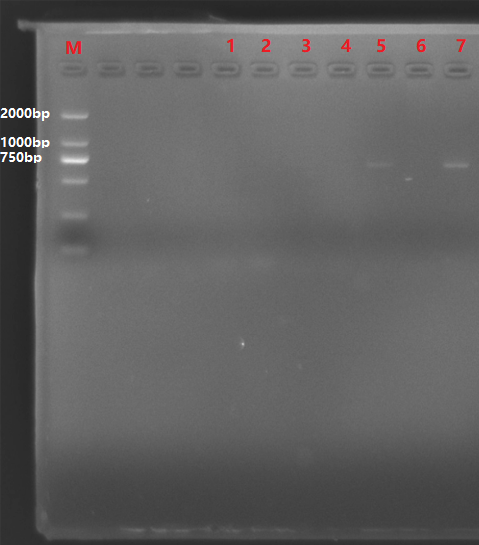

Supplement: Supplementary Figure 1 — PCR-based validation of P. multocida in serum samples. Lanes 1–4, serum samples from the CK-group goats; lanes 5–7, serum samples from the Pm-group goats. [file Image_1.TIF]

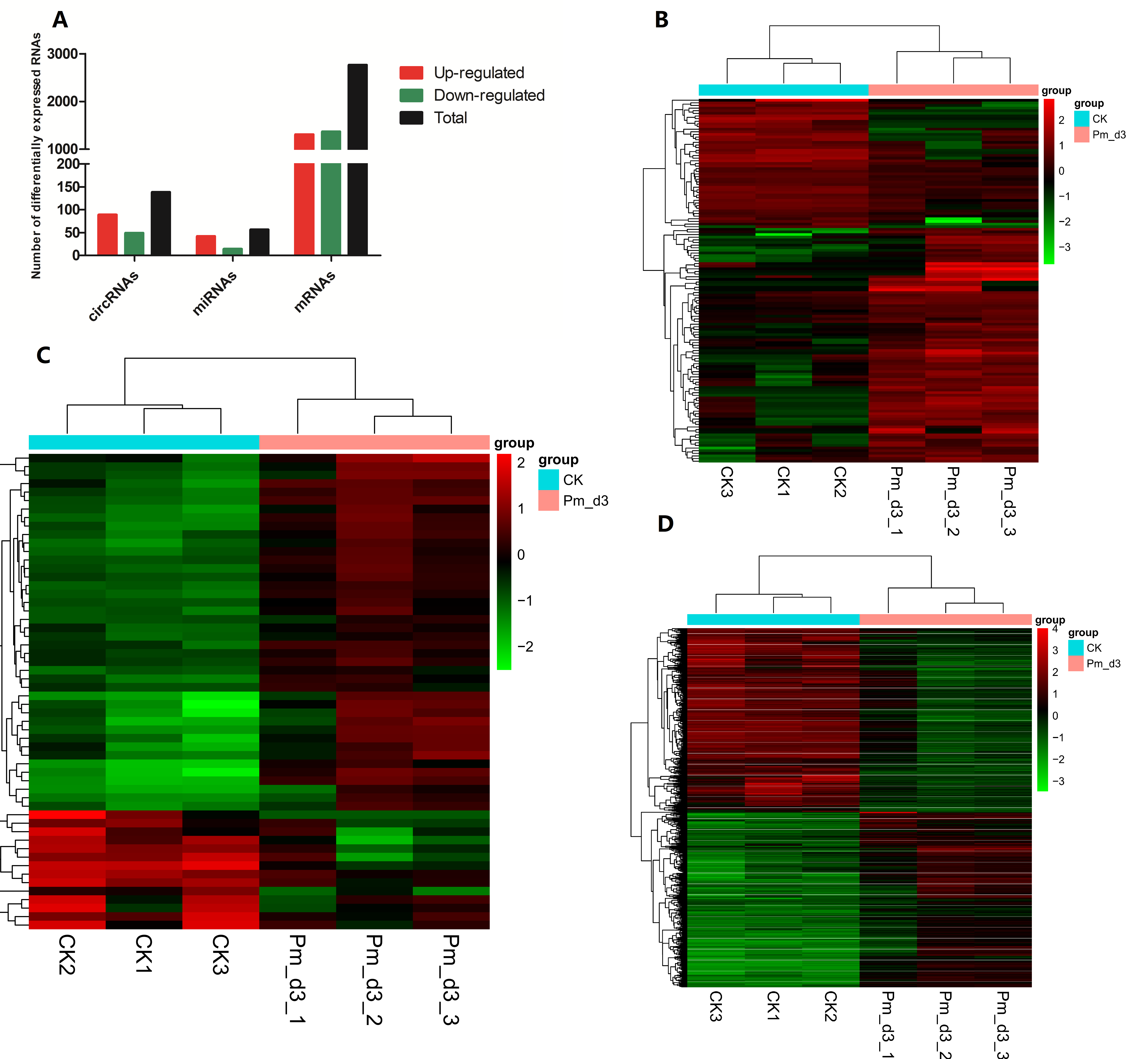

Supplement: Supplementary Figure 2 — Differentially-expressed circRNA, miRNA, and mRNA in the lung tissues of P. multocida-challenged goats compared with the CK group. (A) Numbers of differentially-expressed circRNA, miRNA, and mRNA molecules. (B–D) Hierarchical clustering analysis of differentially-expressed circRNA, miRNA, and mRNA molecules, respectively, identified at p < 0.05 in the goat lung. [file Image_2.TIF]
