# Supplementary material for: Genome-Wide Differential Expression Profiling of Pulmonary circRNAs Associated With Immune Reaction to Pasteurella multocida in Goats
Source: Front Vet Sci. 2021 Jun 21;8:615405. doi: 10.3389/fvets.2021.615405 (PMC8256745; doi:10.3389/fvets.2021.615405)
Supplement: Supplementary Table 1 — Cumulative clinical scoring system. [file Table_1.DOC]

Table S1. Cumulative clinical scoring system.

| Clinical feature | Score | Description |
| --- | --- | --- |
| Rectal temperature (^o^C) | 0 | Normal |
|  | 1 | - |
|  | 2 | ＞40 |
| Respiratory rate (per min) | 0 | ＜20 |
|  | 1 | 20-40 |
|  | 2 | ＞40 |
| Lung auscultation | 0 | Normal |
|  | 1 | Mildly harsh sounds |
|  | 2 | Crackles and wheezes |
| Coughing | 0 | Absent |
|  | 1 | Induced |
|  | 2 | Spontaneous |
| Nasal discharge | 0 | Absent |
|  | 1 | Mild to moderate, serous to mucoid |
|  | 2 | Severe mucopurulent |
| Lacrimation | 0 | Absent |
|  | 1 | Mild to moderate |
|  | 2 | - |
| Conjunctival colour | 0 | Normal |
|  | 1 | Hyperemic |
|  | 2 | - |
